# A national-scale data set for dissolved carbon and its spatial pattern in lakes and reservoirs across China

**DOI:** 10.1038/s41597-020-0419-5

**Published:** 2020-03-09

**Authors:** Zhidan Wen, Kaishan Song, Lili Lyu, Chong Fang, Yingxin Shang, Ge Liu, Jia Du

**Affiliations:** 1 Northeast Institute of Geography and Agroecology, CAS, Changchun, 130102 China; 2 School of Environment and Planning, Liaocheng University, Liaocheng, 252000 China; 3 University of Chinese Academy of Sciences, Beijing, 100049 China

**Keywords:** Environmental sciences, Carbon cycle

## Abstract

A dissolved carbon (DC) data set, including dissolved organic carbon (DOC) and dissolved inorganic carbon (DIC), from 224 lakes (513 stations) and 141 large reservoirs (337 stations) across China is presented in this study. In addition to DC, the data set also includes results for electrical conductivity (EC), total phosphorus (TP), chlorophyll-a and transparency. The impact of trophic status and EC gradient on DC concentration in water bodies are discussed. Results from our investigation indicate that DC in saline (EC > 1000 μS cm^−1^) water bodies (mean ± S.D, 297.13 ± 356.14 mg L^−1^, n = 186) are much higher than those in fresh water bodies (79.55 ± 199.34 mg L^−1^, n = 669). Similarly, eutrophic water bodies (n = 552) exhibited higher DC concentrations than mesotrophic (n = 215) and oligotrophic water bodies (n = 85); DC in lakes (158.445 ± 286.52 mg L^−1^, n = 513) was significantly higher than DC in reservoirs (37.83 ± 37.53 mg L^−1^, n = 337). All data used in this investigation is accessible online.

## Abstract (summary)

**Table:** 

Measurement(s)	dissolved carbon atom in water • electrical conductivity • phosphorus atom • chlorophyll a • water transparency
Technology Type(s)	field survey • conductivity meter • continuous flow analyzer • spectrophotometer • Secchi disk depth
Factor Type(s)	type of water body
Sample Characteristic - Environment	saline water body • fresh water body • eutrophic lake • mesotrophic lake • oligotrophic lake • reservoir
Sample Characteristic - Location	China

Machine-accessible metadata file describing the reported data: 10.6084/m9.figshare.11890596

## Background & Summary

Although inland water bodies occupy a small fraction of the earth’s surface, they play a major role in the global carbon (C) cycle^1^. The predominant terrestrial carbon input to most lakes and reservoirs is dissolved carbon (DC), followed by particulate carbon, and the proportion of inputs varies with lake location and hydrology^2^. The dominant form of aquatic carbon in some temperate regions, high northern latitudes and boreal forests in a carbonate terrain is dissolved inorganic carbon (DIC)^3,4^. In contrast, dissolved organic carbon (DOC) is the dominant form in humid tropical areas and in noncarbonate boreal forests^2^. Spatial DC measurements are useful for understanding carbon sources and cycling in aquatic systems, and it is dependent on an array of factors^2,5,6^, e.g., catchment soil and landscape, hydrology, trophic status and anthropogenic discharge^6–8^. In general, DC in aquatic systems mainly comes from decomposition of organic matter, i.e., plants in the catchment and algae and macrophytes within the water body, and the metabolic secretion of phytoplankton and microbes. However, inland waters in China, particularly those situated in the Northeast, East and South regions, are severely polluted with high nutrients concentration and eutrophic status, which also changes the concentration and constituent of DOC from algae^6,9^. In addition, anthropogenic discharge also provides a large volume of dissolved organic matter into lakes and reservoirs in these regions^10,11^.

Numerous investigations have been carried out to examine the variations in DC in inland waters^5,12–17^. However, investigations of DC patterns are imbalanced with respect to geography^2,5^. Lakes in Europe, particularly in Scandinavian countries where lakes are more abundant, have been intensively investigated^12,18^. Likewise, lakes in the USA, Canada and Japan have also been extensively examined for DC spatiotemporal variation^19–21^. Comparatively, fewer investigations have been undertaken to investigate DC variation for inland waters in China^17^.

Due to differences in climate across the country, and different land-use types surrounding water bodies, China contains a large number of inland water bodies which have a wide variation in water quality^4,22–24^. It was estimated that by 2002, China contained 85,288 reservoirs with a surface water area of 23,000 km^2^, and almost 2700 lakes (area >1 km^2^) with a total area of 81,414.6 km^2^ ^25,26^, with saline lakes accounting for about half of the lake area^27^. Previous investigations have also indicated that DC concentration significantly differs between fresh water and saline water bodies^16,28^. The trophic status of inland water bodies in China ranges from oligotrophic to hypereutrophic. Previous investigations indicated that lakes with eutrophic status in various climatic regions may exert a strong impact on DC of inland waters^6,9,20^. Thus, a systematic examination of DC characteristics in inland water bodies in China is urgently needed.

The objective of this study is to describe a DC data set collected by field surveys undertaken in lakes and reservoirs across mainland China. Our investigation provides a record of DOC and DIC measurements in 288 lakes across five lake regions, and 141 large reservoirs spanning complex topography, landscape, hydrology, eutrophic status, and salinity. Finally, we discuss DC concentrations for lakes and reservoirs with respect to the impact of eutrophic status and a saline gradient in different lake regions in China. We also provide full details for data access.

## Methods

### Study area and lake regions

Surface features in China can be grouped into three levels based on elevation: the first level is represented by the Tibet Plateau; the second level contains areas lying north of the Kunlun Mountains, including several basins and plateaus; the third level consists of hills and plains (Fig. S1). Although climate in China is predominantly dominated by the dry season and the monsoon season, precipitation and temperature patterns across China differ from region to region (Fig. S2). Detailed data for the climate in China is available in Figshare file^29^.

Lake and reservoir density across China vary significantly, largely controlled by terrain, hydrology and population density (Fig. S3a). In addition, reservoirs are also governed by precipitation and geology (Fig. S3b).

**Fig. 1:**
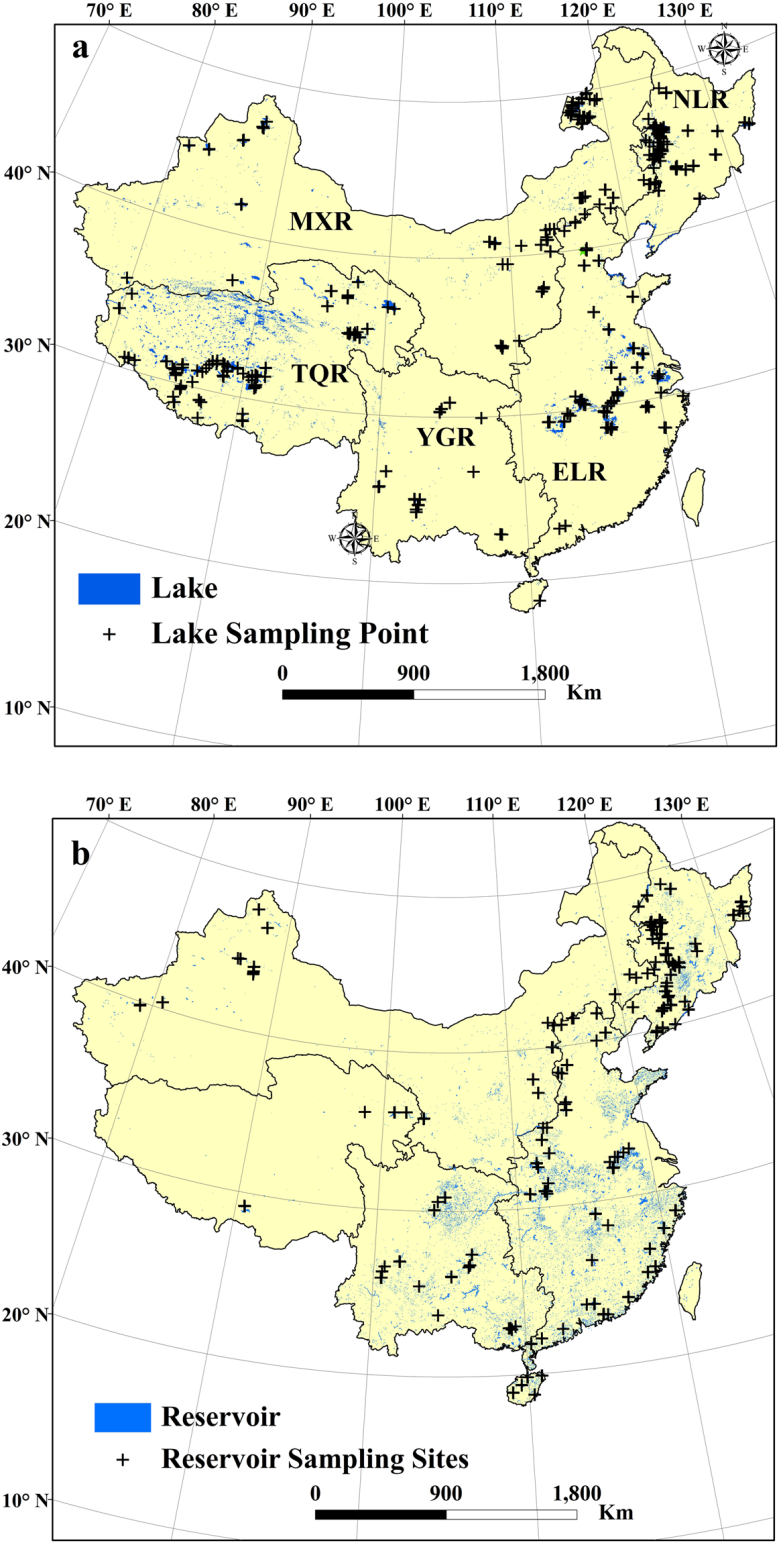
Sampled lakes and reservoirs distribution in mainland of China. (**a**) Lakes distribution and sampled lakes in China, (**b**) reservoirs distribution and sampled reservoirs in China. NLR, northeast limnetic region; ELR, East China limnetic region; MXR, Mengxin limnetic region; YGR, Yungui limnetic region; TQR, Tibet-Qinghai limnetic region.

Lakes in mainland China are divided into five lake regions, according to geography, topography, differences in the natural environment, utilization of lake resources, and the regional characteristics of the overall lake environment^4,22^ (Fig. 1). The five lake regions are: the Northeast Limnetic Region (NLR), East China Limnetic Region (ELR), Inner Mongolia-Xingjiang Limnetic Region (MXR), Yungui Limnetic Region (YGR), and the Tibet-Qinghai Limnetic Region (TQR). In the NLR, lakes are mainly distributed in the Songnen Plain (60%), while reservoirs are mainly situated in the Changbai Mountain, the Daxing’an and the Xiaoxing’an Mountain ranges. In the ELR, lakes and reservoirs are located in the middle and low reaches of fluvial plains formed by the Yellow River, Yangzi River and Huai River. Thousands of closed lakes with a high level of salinity have developed in the TQR, most of which are sensitive to global warming^9^. The total area of lakes and reservoirs with an area greater than 1 km^2^ in each limnetic region are listed in Table S1 ^26^.

### Field surveys and water sampling

Field sampling was undertaken in each limnetic region over varying time periods. In the NLR, 239 samples were collected from 102 water bodies from late September 2012 to October 2017 in 13 field campaigns (Table S1). In the ELR, 241 samples were collected from 56 lakes and 53 reservoirs during six field campaigns, from September 2012 to July 2017. Nine field surveys were undertaken and 152 samples were collected in the MXR; 56 water bodies were sampled in September 2013 and September 2017. In the YGR, two field campaigns were conducted in October 2015 and October 2017, and 78 samples were collected from 32 water bodies in this region. Three field campaigns were carried out in September 2014 (Qinghai Province), June to July 2015 and August to September 2017 (Tibet Autonomous Regions) across the TQR, and 142 samples were collected from 62 lakes and 4 reservoirs. The sampling stations for each lake and reservoir are highlighted on Fig. 1. All lakes and reservoirs were randomly selected, and they were distributed across all major climatic zones in China, as well as in different land-use patterns, including grassland, farmland, forest, desert and artificial land. Lake and reservoir area ranged in size from 0.5 to 4256 km^2^, and both fresh water and saline lakes were included in this study, as well as inflow-outflow lakes and noncontributing lakes.

Water samples were typically collected at 2–3 sampling stations, on average, from every reservoir or lake with a surface area <100 km^2^. If the surface area of the lake/reservoir was >100 km^2^, the number of sampling stations was increased to 5 in each lake/reservoir. Sampling stations were randomly selected away from the shore, and the distance between adjoining points was >2 km. Surface water samples were collected at each station approximately 0.5 m below the water surface. Water samples were collected in amber HDPE bottles and immediately placed into a portable refrigerator at 4 °C before being transported back to the laboratory. All HDPE bottles were acid-washed and rinsed with distilled water before field sampling. Water temperature and total dissolved solids (TDS, in mg L^−1^) were determined using a YSI 600 XLM Sonde with multiple probes (YSI Inc., Yellow Springs, OH). Water transparency was determined using Secchi disk depth (SDD, in meters) for each station for most of the water bodies investigated. All water samples for DOC and DIC analysis were kept in a portable refrigerator at 4 °C for no more than 7 days before they were returned to the laboratory.

A Kruskal-Wallis test was employed to test the difference in the DC concentrations among waters with different trophic status using SPSS 22.0. A Two-Sample Kolmogorov-Smirnov Test was employed as an analysis method using SPSS 22.0 to test the difference in DC concentrations between lakes and reservoirs, between saline and fresh lakes, *p* < 0.05 represents a significant correlation.

## Data Records

### Lake DC concentration

DC concentrations in ELR water bodies ranged from 1.63 to 287.81 mg L^−1^ (median/mean ± SD: 23.65/32.5 ± 38.44 mg L^−1^), indicating a relatively low DC concentration in this region. Lakes in the NLR recorded higher DC concentrations (range of 8.65–2155.25 mg L^−1^), and lakes in the MXR also exhibited a high DC concentration with large variations. Lakes in the YGR recorded average DC concentrations comparable to those in the ELR. A large DC concentration range (2.3–1860.72 mg L^−1^) was recorded for lakes in the TQR, as well as the highest DC concentration (115.66/289.80 ± 408.26 mg L^−1^). This finding was associated to a close link with evapo-concentration for endorheic lakes^16,28^. As shown in Table 1, lakes in the TQR exhibited the highest DC concentrations, followed by MXR and NLR, while water bodies situated in the YGR and ELR displayed lower DC concentrations. Detailed data regarding these results are located in Figshare files^29^.

**Table 1:** Water quality parameters for lakes/reservoirs (L/R) in different limnetic regions across China.

L/R		SDD (L/R)	TP (L/R)	Chl-a (L/R)	DC (L/R)	TSI∑ (L/R)
NLR	Min	0.01/0.1	0.0012/NA	0.09/1.02	8.65/10.86	33.36/38.02
	Max	4.68/3.03	8.44/0.83	459.48/281	2155.25/268.46	87.43/83.81
	Mean	0.9/0.74	0.51/0.07	25.59/27.01	157.59/48.26	61.24/60.9
	SD	1.11/0.7	1.18/0.14	50.91/35.79	257.1/46.09	10.7/10.92
	N	129/167	179/221	182/222	182/222	127/165
ELR	Min	0.12/0.33	0.001/0.001	0.49/0.31	1.63/3.17	40.73/32.37
	Max	2/9.64	1.46/0.47	1262.47/122.81	287.81/158.94	98.83/78.19
	Mean	0.45/2.26	0.15/0.05	35.79/8.69	32.50/24.94	61.74/49.03
	SD	0.34/1.65	0.33/0.06	94.59/14.48	38.44/24.00	9.26/9.41
	N	196/174	103/127	226/191	226/191	87/113
MXR	Min	0.2/0.12	0.004/0.08	0.01/0.17	20.02/7.7	2.51/30.66
	Max	11/4.95	6.31/2.51	139.89/213.88	2108.55/221.35	72.22/74.72
	Mean	1.66/1.43	0.41/0.1	9.84/12.32	184.40/45.1	46.66/46.31
	SD	2.4/1.51	1.07/0.31	18.62/30.4	274.81/39.25	13.86/9.92
	N	127/46	197/70	210/70	211/70	119/46
YGR	Min	0.03/3.03	0.001/0.001	0.59/0.39	21.63/19.36	29.06/22.5
	Max	7.7/11.3	0.19/0.08	1256.19/122.24	193.30/56.14	94.23/61.06
	Mean	2.25/7.1	0.02/0.01	86.81/9.78	45.61/30.51	52.59/44.59
	SD	2.07/3.59	0.04/0.02	197.98/17.74	38.28/6.94	16.48/12.52
	N	94/13	37/26	100/56	100/58	34/20
TQR	Min	0.45/0.4	0.001/0.008	0.01/0.04	2.30/10.50	1.14/19.88
	Max	13.9/5.8	6.79/0.11	31.37/1.4	1830.72/143.09	29.6/29.72
	Mean	5.19/1.89	0.17/0.04	1.67/0.38	289.80/62.56	21.28/28.9
	SD	3.37/1.24	0.63/0.03	3.33/0.29	408.26/55.26	10.73/5.29
	N	90/49	155/23	164/29	169/29	79/9

SDD, Secchi disk depth (meter); Chl-a, chlorophyll-a (μg/L); TP, total phosphorus (mg/L); DC, dissolved carbon (mg/L), TSI, trophic status index, derived from TP, Chl-a and SDD based on Calson’s formulae proposed in 1977 (Limnology & Oceanography, 22(2): 361–369). NLR, northeast limnetic region; ELR, East China limnetic region; MXR, Mengxin limnetic region; YGR, Yungui limnetic region; TQR, Tibet-Qinghai limnetic region.

**Fig. 2:**
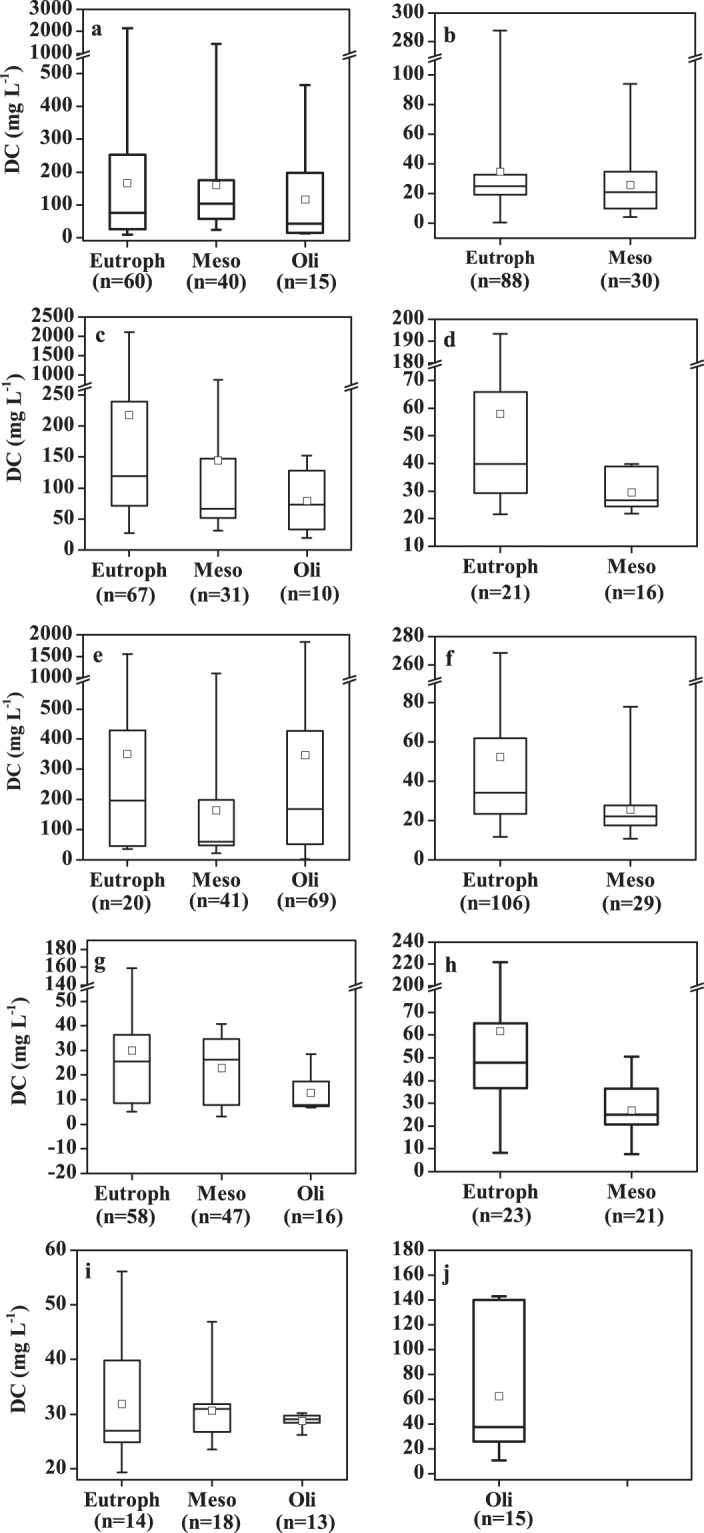
The boxplot of dissolved carbon (DC) in lakes (reservoirs) with different eutrophic status in different lake regions in China. Lakes in (**a**) NLR, (**b**) ELR, (**c**) MXR, (**d**) YGR, and (**e**) TQR; Reservoirs in (**f**) NLR, (**g**) ELR, (**h**) MXR, (**i**) YGR, and (**j**) TQR. Please refer to the caption of Fig. 1 for the abbreviations used for the limnetic regions. The black line and the open square represent the median and mean respectively. The horizontal edges of the boxes denote the 25^th^ and 75^th^ percentiles; the whiskers denote the lower and upper limits of the data.

**Fig. 3:**
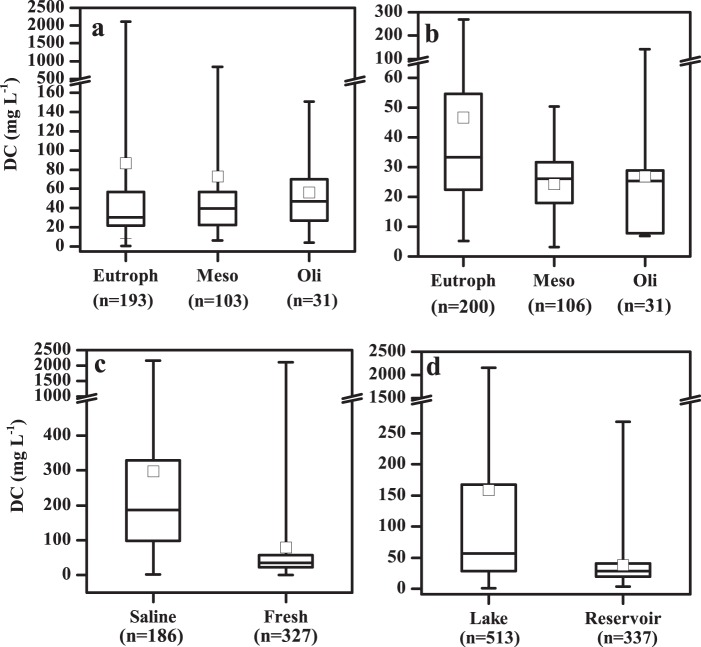
Dissolved carbon (DC) concentrations in sampled inland waters in China. (**a**) In fresh lakes with different trophic status, (**b**) in reservoirs with different trophic status, (**c**) in fresh and saline lakes, (**d**) in lakes and reservoirs. The black line and the open square represent the median and mean respectively. The horizontal edges of the boxes denote the 25^th^ and 75^th^ percentiles; the whiskers denote the lower and upper limits of the data.

Average TSI (Table 1) indicates that most lakes in the NLR and ELR were eutrophic; lakes in the TQR all had an oligotrophic status. As shown in Fig. 2, DC concentrations show considerable variation across lake trophic status. Trophic status is a main regulator of DC in virtually all regions and both system types. In all lake regions, DC concentrations of eutrophic lakes were the highest, and the values in mesotrophic lakes were obviously higher than those in oligotrophic lakes (Fig. 2a–f, *p* < 0.001, *p* = 0.020, *p* < 0.001, *p* < 0.001, *p* = 0.003). This trend was also recorded in the fresh water lake data set when the five lake regions were combined (Fig. 3a, p = 0.02). Detailed data is available in Figshare file^29^.

**Fig. 4:**
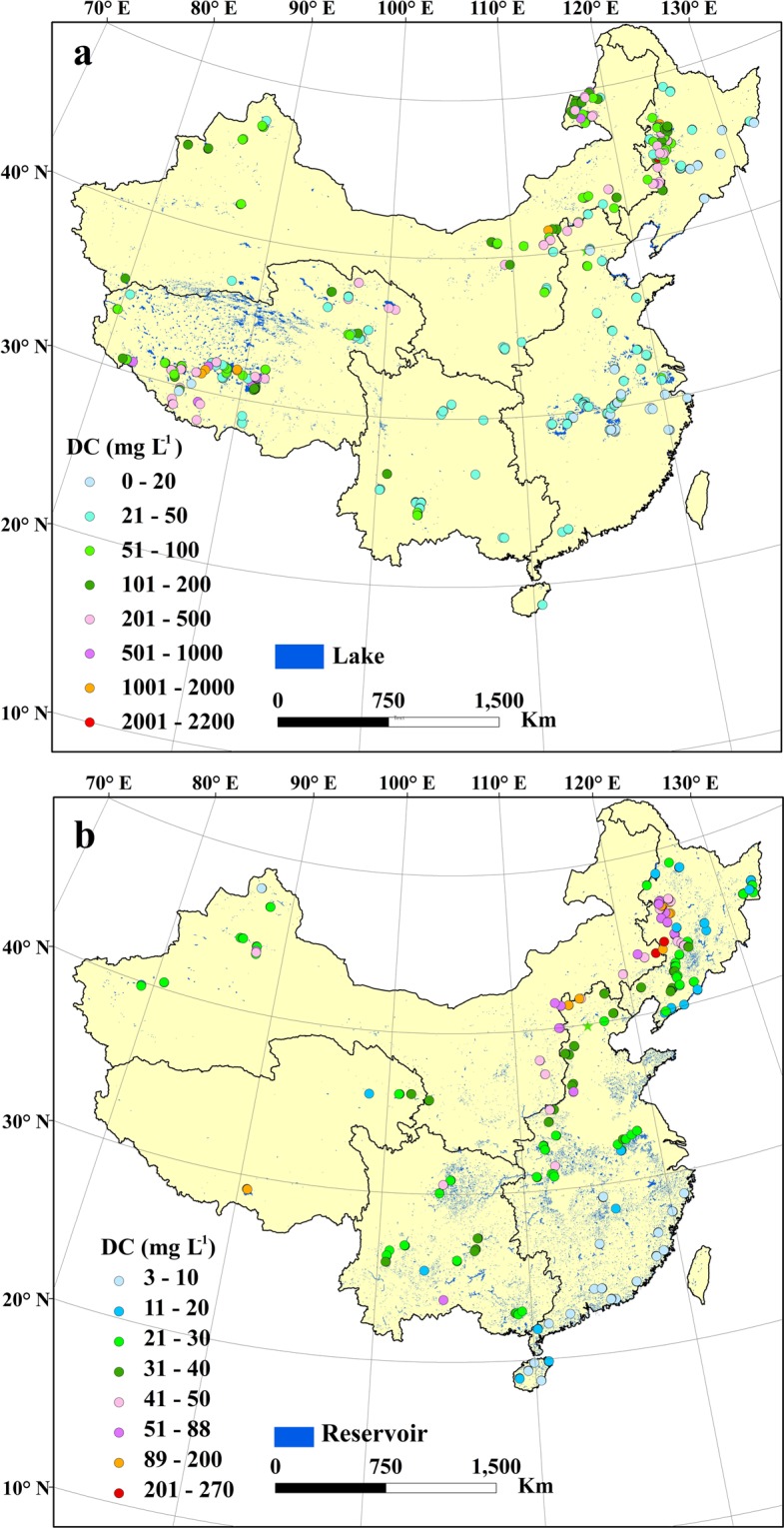
Spatial pattern of dissolved carbon (DC) in waters in China. (**a**) Lake DC spatial pattern, and (**b**) reservoir DC spatial pattern.

DC concentrations in saline water bodies (186.53/297.13 ± 356.14 mg L^−1^, n = 186) were also significantly higher than that in fresh water bodies (35.50/79.55 ± 199.34 mg L^−1^, n = 327) (Fig. 3c, *p* < 0.001) Higher DC concentrations in saline lakes are associated to evapo-concentration effects^6,16,28^. As shown in Fig. S4, endorheic lakes have a wide distribution and DC in these lakes should be further examined. Detailed data is available in file “6. Boundary file for endorheic and five lake regions”. By comparing results in Figs. 2a–e and 4a, higher DC concentrations were found in the NLR and MXR than in other lake regions.

### Lake DOC and DIC concentrations

**Fig. 5:**
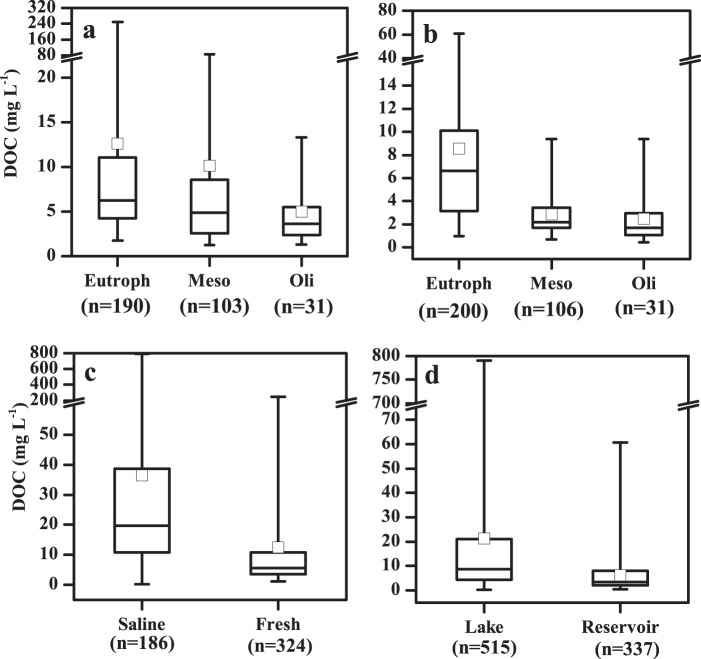
Dissolved organic carbon (DOC) concentrations in sampled inland waters in China. (**a**) In fresh lakes with different trophic status, (**b**) in reservoirs with different trophic status, (**c**) in fresh and saline lakes, (**d**) in lakes and reservoirs. The black line and the open square represent the median and mean respectively. The horizontal edges of the boxes denote the 25^th^ and 75^th^ percentiles; the whiskers denote the lower and upper limits of the data.

**Fig. 6:**
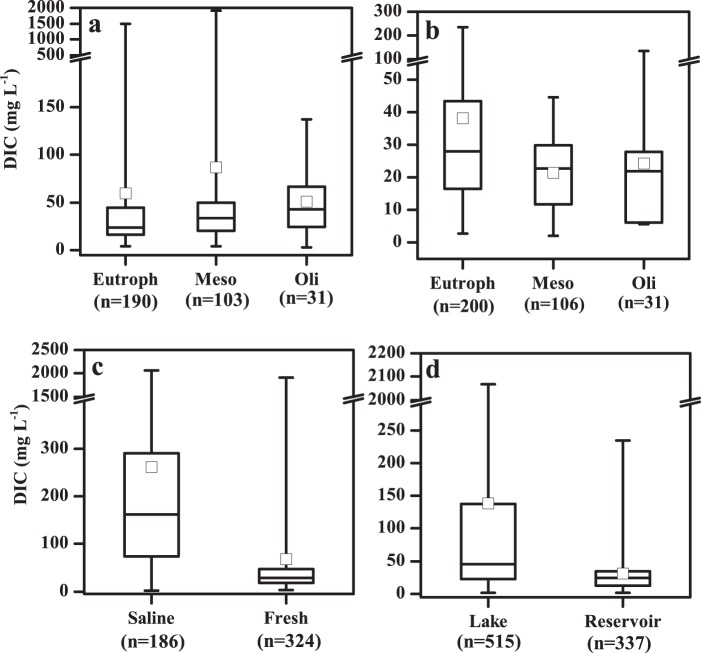
Dissolved inorganic carbon (DIC) concentrations in sampled inland waters in China. (**a**) In fresh lakes with different trophic status, (**b**) in reservoirs with different trophic status, (**c**) in fresh and saline lakes, (**d**) in lakes and reservoirs. The black line and the open square represent the median and mean respectively. The horizontal edges of the boxes denote the 25^th^ and 75^th^ percentiles; the whiskers denote the lower and upper limits of the data.

Lakes in the MXR recorded the highest DOC concentration (25.9/32.8 ± 53.4 mg L^−1^), followed by TQR, NLR and ELR; lakes in the YGR recorded the lowest DOC concentration (3.5/5.4 ± 4.4 mg L^−1^). DOC concentrations in fresh lakes significantly differed with trophic status: DOC concentrations of eutrophic lakes were the highest, and concentrations in mesotrophic lakes were notably higher than those in oligotrophic lakes (Fig. 5a, p < 0.001). DOC concentrations in saline water bodies were significantly higher than those in fresh water bodies (Fig. 5c, p < 0.001). The main reason for higher DOC concentrations in saline lakes was due to the evapo-concentration effect^16,28^. DIC concentrations in fresh lakes also differed as the trophic status changed (Fig. 6a, p = 0.006), and DIC in saline water bodies was significantly higher than that in fresh water bodies (Fig. 6c, p < 0.001). Detailed data information highlighting these findings are available in Figshare file^29^.

### Reservoir DC concentration

Average DC concentrations in reservoirs in the MXR (Fig. 2h) are comparable to those in the NLR; reservoir results in the ELR recorded a relatively low average DC concentration (Fig. 2g). DC concentrations in reservoirs in the NLR and in the eastern area of the MXR were higher than those in other regions (Fig. 4b). Our results indicate that eutrophication also exerts a strong correlation with DC concentrations in reservoirs in different regions across China (Fig. 2f–i). DC concentrations versus trophic status notably differed, and the general trend for DC concentrations in different trophic status exhibited a similar pattern for lakes. However, the impact of eutrophication on reservoirs in the TQR were not identified, and DC concentration is more relevant to salinity. The impact of eutrophication on DC concentration is also highlighted in the pooled data set with reservoirs (Fig. 3b). In addition, our data set indicated that DC in lakes (56.8/158.44 ± 286.52 mg L^−1^, n = 513) was significantly higher than that in reservoirs (27.84/37.83 ± 37.53 mg L^−1^, n = 337) (*p* < 0.001) (Fig. 3d). Detailed data for this finding is present in Figshare file^29^.

### Reservoir DOC and DIC concentration

Average DOC concentrations in reservoirs in the NLR and the eastern area of the MXR were higher than those in other regions; reservoirs in the YGR recorded relatively low average DOC concentrations. DOC concentrations versus trophic status were notably different, and the general trend for DOC concentrations in different trophic status’ exhibited a similar pattern in lakes (Fig. 5b, p < 0.001). However, where DOC concentration was linked to salinity, the impact of eutrophication on reservoirs in the TQR was not evident. Furthermore, results indicated that DOC in lakes (7.6/20.0 ± 46.7 mg L^−1^, n = 513) was significantly higher than DOC in reservoirs (3.7/6.5 ± 7.6 mg L^−1^, n = 337) (Fig. 5d, p < 0.001). DIC concentrations in reservoirs also differed as trophic status changed (Fig. 6b), and DIC in lakes was significantly higher than that in reservoirs (Fig. 6d, p < 0.001).

## Technical Validation

### DC measurements

Water quality parameters were measured within 24 h after arriving in the laboratory. To determine dissolved carbon (DC), water samples were initially filtered through pre-combusted 0.45 μm glass-fiber filters (Bandao Industrial Co., Ltd, China) before analysis using a Total Organic Carbon Analyzer (Shimadzu, TOC-VCPN). All water samples were measured using the same instrument with the same protocol for DC determination. In addition, results from our previous study indicated that DC concentrations for some saline lakes exceeded 100 mg L^−1^ ^6,17^. Samples exceeding this limit were diluted using Milli-Q water to ensure concentrations were within the measurement range of the analyzer (TOC analyzer had a measuring range of 0–100 mg L^−1^). Analysis of blanks and replicates showed a detection limit of 0.3 mg L^−1^ and a precision of 5% at a concentration of 4 mg L^−1^.

### Water quality determination

In the laboratory, electrical conductivity (EC, μS cm^−1^) was measured at room temperature (20 ± 2 °C) using a DDS-307 EC meter. Chlorophyll-a (Chl-a) was extracted from water samples using a 90% buffered acetone solution, and the concentration for each water sample was determined using a Shimadzu UV-2660 PC spectrophotometer^30^. Total phosphorus (TP) concentration in water samples was determined using a Continuous Flow Analyzer (SKALAR, San Plus System, Netherlands), using the molybdenum blue method after samples had been digested using potassium peroxydisulfate^31^.

### DC regulating factors

The eutrophic status for lakes and reservoirs in China were classified into oligotrophic, mesotrophic and eutrophic water bodies according to SDD, Chl-a and TP based on the Carlson Trophic Status Index (TSI)^32,33^. This method used a series of consecutive numbers (0–100) to represent the nutrient classification state of the lakes: TSI ≤ 30 indicated an oligotrophic state, (30–50] indicated a mesotrophic state, and (50–100] indicated a eutrophic state. In addition, as DC concentration in lakes also has a strong association with salinity^6,16,28^, DC in lakes across China were characterized as brackish or fresh water bodies using EC > 1000 μS cm^−1^.

## Supplementary information

Supporting Information

## Data Availability

No custom code was used in the study.
