# Supplementary material for: A national-scale data set for dissolved carbon and its spatial pattern in lakes and reservoirs across China
Source: Sci Data. 2020 Mar 9;7:82. doi: 10.1038/s41597-020-0419-5 (PMC7062785; doi:10.1038/s41597-020-0419-5)
Supplement: Supplementary file 1 — Supporting Information [file 41597_2020_419_MOESM1_ESM.docx]

**Supporting Information**

**A national-scale data set for dissolved carbon and its spatial pattern in lakes and reservoirs across China**

Zhidan Wen^1^†, Kaishan Song^1, 2^†*, Lili Lyu^1^, Chong Fang^1, 3^, Yingxin Shang^1, 3^, Ge Liu^1^, Jia Du^1^

1. Northeast Institute of Geography and Agroecology, CAS, Changchun 130102, China

2. School of Environment and Planning, Liaocheng University, Liaocheng 252000, China

3. University of Chinese Academy of Sciences, Beijing 100049, China

**Contents of this file:**

| **Contents** | **Page** |
| --- | --- |
| Figure S1 The distribution of lakes and reservoirs with elevation gradients based on DEM (digital elevation model) in mainland of China | 1 |
| Figure S2 The spatial pattern of precipitation (a) and temperature (b) in different limnetic regions across mainland of China | 2 |
| Figure S3 Water bodies in mainland of China delineated through Landsat images acquired in 2015-2016, (a) lake distribution, and (b) reservoir distribution across mainland of China | 3 |
| Figure S4 Endorheic versus outflow lakes (exoreic) across mainland of China | 4 |
| Table S1 Field surveys conducted over lakes and reservoirs of different limnetic regions across mainland of China | 5 |


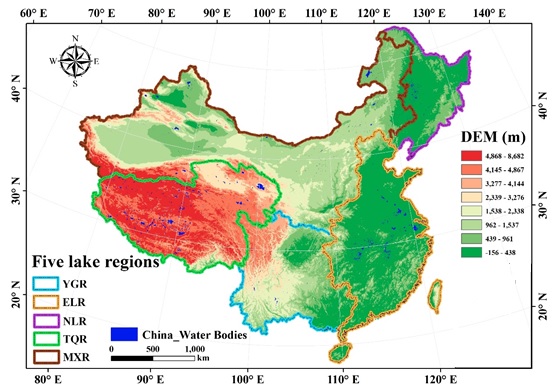


Figure S1 The distribution of lakes and reservoirs with elevation gradients based on DEM (digital elevation model) in mainland of China. YGR, Yungui limnetic region; ELR, East China limnetic region; NLR, Northeast China limnetic region; TQR, Tibet-Qinghai limnetic region; MXR, Inner Mongolia-Xingjiang Limnetic Region.


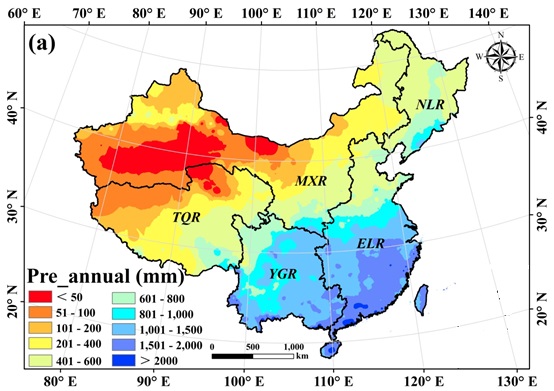


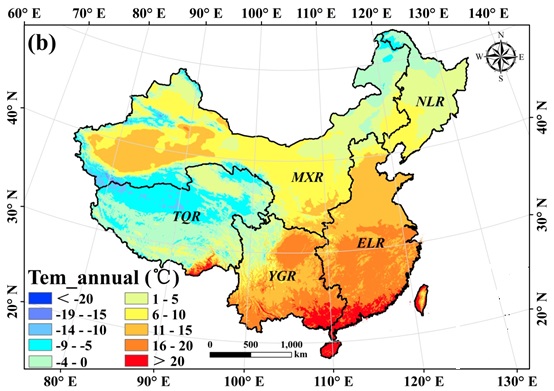


Figure S2 The spatial pattern of precipitation (a) and temperature (b) in different limnetic regions across mainland of China.


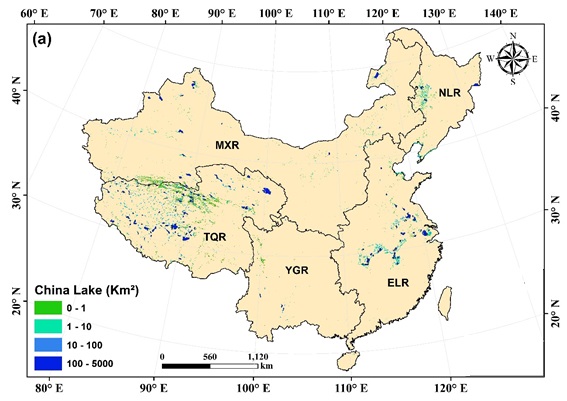


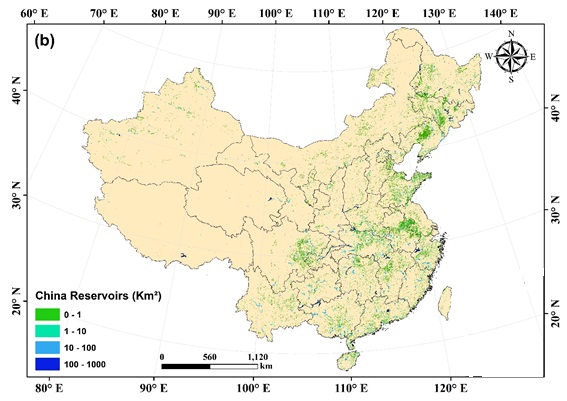


Figure S3, Water bodies in mainland of China delineated through Landsat images acquired in 2015-2016, (a) lake distribution, and (b) reservoir distribution across mainland of China.


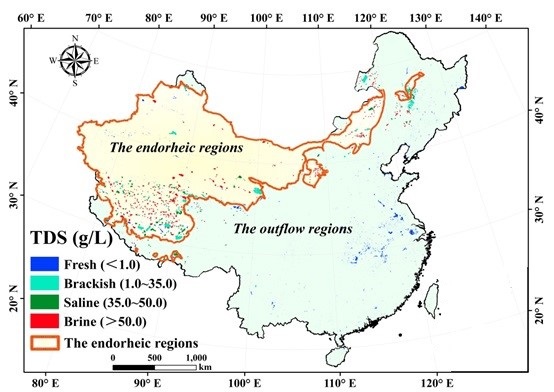


Figure S4 Endorheic versus outflow lakes (exoreic) across mainland of China

Table S1 Field surveys conducted over lakes and reservoirs of different limnetic regions across mainland of China

| Limnetic Region | Lakes | L.S. | Reservoirs | R.S. | Sampling time (Year/month/date) |  |
| --- | --- | --- | --- | --- | --- | --- |
| NLR | 57 | 114 | 45 | 125 | 2012/09/21,2012/09/22  2013/10/11,2014/10/28  2015/07/06-2015/07/08  2015/08/15,2015/08/21  2015/09/14-2015/09/18  2016/04/11-2016/04/29  2017/06/26-2017/06/28  2017/08/30  2017/09/26-2017/10/14  2017/10/29-2017/10/30 |  |
| ELR | 56 | 120 | 53 | 121 | 2014/10/10-2014/10/12  2015/09/30-2015/11/06  2017/05/06  2017/06/25-2017/07/06 |  |
| MXR | 35 | 108 | 21 | 44 | 2013/09/06,2013/09/09  2013/10/11  2014/07/16-2014/07/17  2015/07/06-2015/07/20  2015/09/14-2015/09/18  2015/10/10  2017/04/21-2017/05/05  2017/09/08 |  |
| YGR | 14 | 43 | 18 | 35 | 2015/10/16-2015/10/27  2017/10/19-2017/10/20 |  |
| TQR | 62 | 130 | 4 | 12 | 2014/09/14-2014/09/15  2015/06/21-2015/07/06  2017/08/15-2017/09/05 |  |

The numbers in the Lakes and Reservoirs column represent the number of sampling lakes or Reservoirs; L.S., the total number of sampling stations in lakes; R.S., the total number of sampling stations in reservoirs.
